# Systematic Characterisation of GLP‐1R in Human Enteric Nervous System: Implications for GLP‐1 as a Key Regulator of Colonic Activity

**DOI:** 10.1111/jnc.70461

**Published:** 2026-05-15

**Authors:** Kiran Devi Dontamsetti, Rubina Aktar, Madusha Peiris

**Affiliations:** ^1^ Wingate Institute of Neurogastroenterology, Blizard Institute, The Faculty of Medicine and Dentistry Queen Mary University of London London UK

**Keywords:** enteric nervous system, gastrointestinal motility, GLP‐1R, human gastrointestinal tract, neurochemical coding

## Abstract

Glucagon‐like peptide‐1 (GLP‐1) regulates glucose homeostasis, satiety and gastrointestinal (GI) motility through interaction with its receptor (GLP‐1R). While central GLP‐1 pathways are well studied, the distribution and functional role of GLP‐1R within the human enteric nervous system (ENS) remain unclear. We characterised GLP‐1R expression across the human GI tract, examining its anatomical localisation among distinct enteric neuronal subtypes. Non‐inflamed, full‐thickness human GI tissues (antrum, ileum, ascending, descending and sigmoid colon; *N* = 30, 5 different human samples per region) were obtained from surgical resections and analysed using immunohistochemistry, epifluorescence and confocal microscopy. Mean number of positive pixels of GLP‐1R and PGP9.5 was quantified in mucosal varicosities, muscle, submucosal and myenteric plexuses. GLP‐1R co‐localisation with neuronal markers (PGP9.5, nNOS, ChAT, Substance P, CGRP, Calretinin, HuC/D) was assessed. Quantification used Mander's coefficient, and statistical analysis used one‐way ANOVA with Tukey's post hoc test. GLP‐1R was expressed abundantly in ENS structures, including mucosal varicosities, muscle, submucosal plexus and myenteric neurons throughout the lower GI tract, with significantly higher expression in the colon compared to the stomach and ileum (*p* < 0.05). Co‐localisation analyses revealed preferential GLP‐1R expression in nNOS‐expressing inhibitory neurons and CGRP‐expressing varicosities, moderate expression in calretinin‐expressing neurons, and sparse expression in ChAT‐expressing and substance P‐immunoreactive excitatory neurons. Whole‐mount confocal imaging confirmed GLP‐1R localisation to HuC/D‐immunoreactive neuronal cell bodies with punctate, membrane‐associated staining. GLP‐1R is differentially expressed across the human ENS, with increased expression in inhibitory neurons and putative extrinsic sensory afferents, particularly in the distal colon. This suggests that peripherally released GLP‐1 acts locally within the ENS to regulate motility and sensory signalling, underpinning both its physiological functions and the gastrointestinal side effects of GLP‐1‐based therapies.

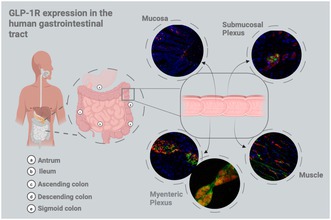

## Graphical abstract

Glucagon‐like peptide‐1 (GLP‐1) regulates glucose homeostasis, appetite and gastrointestinal motility through activation of the GLP‐1 receptor (GLP‐1R). Although central mechanisms are well established, the anatomical distribution of GLP‐1R within the human enteric nervous system (ENS) has not been fully defined. Here, we map GLP‐1R expression across the human gastrointestinal tract and demonstrate widespread localisation within enteric neural compartments, including mucosal nerve fibres, submucosal and myenteric plexuses, and smooth muscle layers, with enrichment in the distal colon. GLP‐1R preferentially localises to inhibitory (nNOS‐positive) enteric neurons and CGRP‐immunoreactive sensory varicosities, with comparatively limited expression in excitatory cholinergic and substance P–positive neuronal populations. These findings support a model in which peripherally released GLP‐1 directly modulates enteric neural circuits, particularly inhibitory motor and sensory pathways, providing a mechanistic basis for its effects on gastrointestinal motility and the gastrointestinal actions of GLP‐1–based therapies.

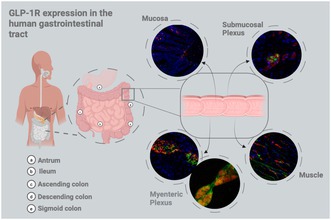

**Abbreviations**
AOI: area of interest
Asc: ascending colon
CalRet: calretinin
CGRP: calcitonin gene‐related peptide
ChAT: choline acetyltransferase
DAPI: 4′,6‐diamidino‐2‐phenylindole
Desc: descending colon
ENS: enteric nervous system
F: female
FOV: field of view
GLP‐1: glucagon‐like peptide‐1
GLP‐1R: glucagon‐like peptide‐1 receptor
IBD: inflammatory bowel disease
IHC: immunohistochemistry
M: male
nNOS: neuronal nitric oxide synthase
PBS: phosphate‐buffered saline
RRID: research resource identifier (see scicrunch.org)
Sig: sigmoid colon
SP: substance‐P
T2DM: type 2 diabetes mellitus

## 1 Introduction

Both central and peripheral mechanisms regulate appetite and food intake (Browning et al. 2017; Cifuentes and Acosta 2022). Peripherally, gut hormones are secreted by specialised enteroendocrine cells distributed along the human gastrointestinal (GI) tract (Gribble and Reimann 2016; Symonds et al. 2015; Yang et al. 2021). These gut hormones—including glucagon‐like peptide 1 (GLP‐1), PYY and ghrelin—communicate vital information about nutrient availability, glucose homeostasis, and overall energy status to the central nervous system (CNS). Notably, GLP‐1 secreted by L‐cells has gained increasing recognition for its physiological importance in modulating appetite, insulin secretion and motility (Symonds et al. 2015; Page et al. 2012; Tolhurst et al. 2008).

GLP‐1 is synthesised in two distinct anatomical locations within the body: the preproglucagon‐expressing neurons of the brain and the enteroendocrine L‐cells of the GI tract. In the CNS, GLP‐1 is transported to the axon terminals of these neurons and released in the hypothalamus and spinal cord (Drucker 2001). Although both central and peripherally derived GLP‐1 act on the same GLP‐1 receptor (GLP‐1R), which is widely expressed throughout the body, the functional distinctions between centrally and peripherally derived GLP‐1 remain unclear. Peripheral signals converge to influence feeding behaviours and energy expenditure (Yoo et al. 2021).

Within the hypothalamus, the arcuate nucleus (ARC) contains pro‐opiomelanocortin (POMC) anorectic neurons and neuropeptide Y/agouti‐related peptide (NPY/AgRP) incretin neurons (Xu et al. 2011). These ARC networks communicate with other hypothalamic regions, notably the paraventricular nucleus (PVN), which together coordinate energy homeostasis (Williams et al. 2009; Williams 2009; Sandoval et al. 2008; Näslund et al. 1999; Holst et al. 2022).

Experimental studies indicate that blocking ARC GLP‐1 receptors results in a 50% increase in the glycaemic response to intraperitoneal glucose compared to saline‐infused rats (Sandoval et al. 2008). Additionally, in both humans and rodents, acute GLP‐1 administration reduces caloric intake and increases satiety (Näslund et al. 1999). Nevertheless, the predominant endogenous source of circulating GLP‐1 is the intestinal L‐cell, underlying the importance of understanding peripheral GLP‐1R expression and function within the human GI tract.

Peripherally produced GLP‐1, synthesised by L‐cells, exists in two forms: GLP‐1‐_7‐37_ and GLP‐1‐_7‐36_ and has an important role in local GI physiology (Drucker 2001; Holst et al. 2022). However, GLP‐1‐_7‐36_ has a short half‐life of 1.5 to 5 min due to rapid enzymatic degradation by dipeptidyl peptidase IV (DPP‐IV) (Gilbert and Pratley 2020). Despite this, active GLP‐1 significantly influences gut physiology, including slowing gastric emptying, reducing gastrin‐induced acid secretion and regulating antro‐duodenal motility (Schirra et al. 2006; Willms et al. 1996; Kreymann et al. 1987). Physiologically, GLP‐1 activates the ileal brake, inhibiting gastric emptying and small intestinal transit (Tolessa et al. 1998a; Halim et al. 2018).

These gut functions are controlled by neural signals within the gut, regulated by the ENS. Intrinsic activity of the ENS regulates site‐specific activities such as secretion and peristalsis, while extrinsic pathways modulate gut motility and appetite via bidirectional gut‐brain axis signalling (Rao and Gershon 2016). These motor and secretory functions of the ENS/gut are primarily mediated by ganglionated plexuses embedded in the gastrointestinal wall (Furness 2012; Furness et al. 2014). The submucosal plexus, positioned between the mucosa and submucosa, regulates glandular secretion, blood flow and local muscular contraction (Furness et al. 2014). In contrast, the myenteric plexus, located between the circular and longitudinal muscle layers, primarily coordinates GI motility (Furness 2012; Furness et al. 2014; Maqbool 2013; Forsythe et al. 2014). In humans and large animals, motor neurons in the submucosal plexus innervate the muscularis externa along with the well‐known innervation from myenteric neurons, while in all species, there is bidirectional crosstalk between the myenteric plexus and submucosal plexus, resulting in coordinated secretion and motility (Furness et al. 2014).

In addition to local GI physiology, GLP‐1 plays a vital trophic role in pancreatic β cells as an incretin hormone. In some cases where dual standard therapies fail, GLP‐1 receptor agonists are utilised as treatment for type 2 diabetes mellitus (T2DM) (Gilbert and Pratley 2020; Sun et al. 2022; Sharma et al. 2018). While GLP‐1 agonists were initially developed to improve insulin levels in T2DM management, their use as weight‐loss treatments is growing rapidly (Mailhac et al. 2024). Widespread use of GLP‐1R mimetics as anti‐obesity medications highlights the prevalence of GI adverse effects, with 25%–60% of treated patients reporting nausea, 5%–15% experiencing vomiting, and 10%–20% suffering from diarrhoea while on semaglutide and tirzepatide treatment (Sharma et al. 2018; Nauck et al. 2021; Fahim et al. 2025). These adverse effects are attributed to GLP‐1's impact on post‐prandial insulin secretion, which reduces peristalsis (Holst et al. 2022; Nauck et al. 2021). However, there is an urgent need to better understand gut‐specific GLP‐1R signalling pathways.

Beyond GLP‐1R's well‐defined role in pancreatic and central nervous system signalling, accumulating evidence indicates that GLP‐1Rs are also expressed within the ENS. GLP‐1R expression has been identified in a subset of murine enteric neurons, including cholinergic and nitrergic enteric neurons (Amato et al. 2010). However, precise co‐localisation with defined neurochemical markers in human tissues remains unresolved. Within the human GI tract, GLP‐1Rs are present in the bowel, where GLP‐1R agonists delay gastric emptying (Halim et al. 2018) whilst nutrient ingestion, such as oral glucose, stimulates GLP‐1 release (Kreymann et al. 1987). At the molecular and histological levels, GLP‐1R is found in human gastric glands (Broide et al. 2013) and in inflammatory profiles such as inflammatory bowel disease (IBD), where there is increased GLP‐1R colocalisation on sensory neuropeptide‐CGPR expressing nerve fibres (Anand et al. 2018).

Overall, gut hormones like GLP‐1 are intricately linked to nutrient intake‐mediated regulation of gut activity to promote digestive processes (Symonds et al. 2015; Dontamsetti et al. 2024; Peiris et al. 2022). Despite extensive research on GLP‐1's central effects, its exact mechanism of action in peripheral tissues remains poorly characterised, particularly in human tissue, as most data are from animal studies. To address this gap, we characterised GLP‐1R expression across the human GI tract.

## 2 Methods

This was an exploratory study which was not pre‐registered.

### 2.1 Tissue Collection

Tissue was collected from 30 patients undergoing GI surgery at the Royal London Hospital (Barts and the London School of Medicine and Dentistry, Queen Mary University of London, UK), with prior informed consent (East London and The City HA Local Research Ethics Committee (NREC 09/H0704/2)). Surgically resected human tissues were collected from the antrum, ileum, ascending, descending, and sigmoid colon. Non‐pathological full‐thickness tissue samples were dissected by a trained histopathologist, with a margin of 10 cm proximally from any tumour, and all tissues were macroscopically uninflamed. Tissue was taken from 16 females and 14 males; average age: 60 ± 8 years. Further patient demographic information can be found in Tables S1 and S2.

Tissue was placed in ice‐cold carbogenated Krebs solution (NaCl; 124.05 mmol/L, KCl; 4.78 mmol/L, CaCl_2_; 2.5 mmol/L, MgSO_4_; 2.44 mmol/L, NaHCO_3_; 25 mmol/L, NaH_2_PO_4_; 1.33 mmol/L, D‐Glucose; 5.5 mmol/L, bubbled with 95% O_2_, 5% CO_2_, pH; 7.4) and transported to the laboratory.

Full‐thickness tissue was immediately fixed in 4% paraformaldehyde overnight (Sigma‐Aldrich, Cat. Number 158127). Following fixation, tissue was washed in PBS, cryoprotected in 30% sucrose/phosphate‐buffered saline overnight at 4°C, then transferred to a 50% optimal cutting temperature (OCT; Sakura Tissue‐Tek)/50% sucrose solution for 16–18 h at 4°C. Before embedding in OCT, tissue was snap‐frozen and stored at −20°C. Serial histological transverse sections were cut using a Cryostat (Leica, CM1860 UV) at a thickness of 10 μm and mounted on positively charged glass slides (Thermo Scientific, Cat. Number J1800AMNZ).

### 2.2 Immunohistochemistry (IHC) Process for Sections

For immunohistochemistry (IHC), positively charged slides with 10 μm‐thick sections were rehydrated in PBS for 5 min at room temperature. Sections were blocked using protein‐block (Protein Block Serum‐Free Ready‐to‐use, Dako, Cat. Number X0909) for 1 h at room temperature in the dark. Sections were incubated with a primary antibody solution containing PBS and the detergent 0.2% Triton X‐100 for 16–18 h. All antibodies used are described in Table 1. After overnight incubation, the slides were washed in PBS and incubated with secondary antibodies for 1 h at room temperature (donkey anti‐mouse 568 nm, donkey anti‐rabbit 488 nm; Invitrogen, Thermo Fisher Scientific, 1:400 dilution). They were then washed in PBS whilst being gently agitated. Slides were mounted with DAPI fluorescent mounting stain (Vector Laboratories, Cat. Number H‐1500) and a cover glass. Slides were imaged using a Leica DM5000B Epi‐Fluorescence Microscope, and images captured using Cell Sens microscopy software. Images were viewed at 40× objective and processed using FIJI software. For uniformity, all sections were oriented and cut in the same serial manner. Per anatomical region, samples from five different patients were assessed. The specificity of all immunostainings was confirmed with negative controls and no primary antibodies (Figures S1 and S2).

**TABLE 1:** Antibodies used for IHC. The primary antibodies are listed here, along with the concentrations used to achieve an optimal signal‐to‐noise ratio.

	Species	Concentration fixed frozen	Concentration whole mount	Source	Code	RRID
**Primary antibody**						
*GLP‐1R*	Rabbit	1:400	1:200	Alamone	AGR‐021	AB_10917158
*PGP9.5*	Mouse	1:200	1:200	Biotechne	MAB60072	AB_3658518
*Calretinin*	Goat	1:400		Swant	CG1	AB_10000342
*Substance P*	Rat	1:400		Santa Cruz	SC‐21715	AB_628299
*ChAT*	Goat	1:200		Bio‐Techne	NBP130052	AB_2079586
*nNOS*	Goat	1:200		GeneTex	GTX89962	AB_10725945
*CGRP*	Mouse	1:200		ThermoFisher	PA5114929	AB_2899565
*Huc/D*	Mouse	1:200	1:200	Invitrogen	21 271	AB_221448
**Secondary Antibody**						
*AF‐488*	Donkey‐Rabbit	1:400	1:300	Invitrogen	A21206	AB_2535792
	Donkey‐Mouse	1:400	1:300	Invitrogen	A21202	AB_141607
	Donkey‐Goat	1:400		Invitrogen	A11055	AB_2534102
	Donkey‐Rat	1:400		Invitrogen	A21208	AB_2535794
*AF‐568*	Donkey‐Mouse	1:400	1:300	Invitrogen	A10037	AB_11180865
	Donkey‐Rabbit	1:400	1:300	Invitrogen	A10042	AB_2534017

### 2.3 Wholemount IHC of Myenteric Plexus

Full‐thickness tissue from the resected sigmoid colon was dissected to separate and remove mucosa and serosa, to expose the muscularis externa. Muscle layers were separated using a small incision with closed fine scissors, to slowly separate fibres. Remaining connective tissue and muscle were removed with a scalpel, and the cleaned preparation was pinned under tension onto an agar support strip to maintain orientation. Following dissection, the muscle strips were transferred into 1.5 mL Eppendorf tubes and fixed in 4% paraformaldehyde (PFA) for 2 h with agitation (Sigma‐Aldrich, Cat. Number 158127).

After fixation, the samples were washed in a blocking solution containing protein block and 0.2% Triton X‐100 in PBS for 12–16 h at 4°C under continuous agitation, whilst pinned under tension. Subsequently, the muscle strips were incubated overnight at 4°C in primary antibody solutions (concentrations listed in Table 1). Samples were then washed overnight at 4°C under continuous agitation with tension and incubated with appropriate secondary antibodies for 3 h at room temperature whilst agitated. Finally, the muscle strips were washed for 3 h in PBS, with PBS changes every 30 min. From each muscle strip, a sample was isolated to prepare whole mounts of the myenteric plexus and associated enteric neurons. After additional PBS washes, preparations were mounted on glass slides (Fisher UK, 10354365) with coverslips added (Avantor Cat. No 631‐0137) and secured with thread. Wholemounts were imaged using a Zeiss LSM 880 Laser Scanning Confocal Microscope (Zeiss, Oberkochen, Germany, RRID:SCR_020925), and Z‐stack images were acquired at optimal intervals (0.341 μm) between optical slices.

### 2.4 Image Analysis

Images from full‐thickness sections were taken from separate areas of interest (AOI), specifically, mucosa, submucosal plexus, myenteric plexus, and muscle layers. A minimum of five fields of view (FOVs) per patient/region were captured for all sections. To quantify the receptor‐positive area, individual fluorescence channels were separated and converted to 8‐bit greyscale images. A global threshold was applied using Otsu threshold to distinguish specific signal from background. The receptor‐positive area was calculated by multiplying the percentage above threshold by the total region of interest and dividing by 100 to give the absolute area of receptor‐positive signal. The area of interest was selected using the freehand tool based on the DAPI stain.

Images with positive staining were processed using Otsu's threshold to segment areas of interest. Mean number of positive pixels within these areas of interest was quantified using FIJI (Fiji Is Just ImageJ) software (RRID:SCR_002285). Co‐localisation was measured using the JaCoP (Just another Co‐localisation Plugin) macro from FIJI to calculate the Manders' coefficient (M_1_ and M_2_). Images were converted from 16‐bit to 8‐bit using the batch convert tool. Images were then split, using the split channels tool, for the GFP (GLP‐1R Antibody) and RFP (PGP9.5) images. Mander's coefficient calculated the proportion of one fluorescent signal overlapped with another, that is, GLP‐1R, overlap with PGP9.5 or neuronal antibodies. Image A was assigned to GLP‐1R staining, and Image B was assigned to PGP9.5. M_1_ and M_2_ values were calculated, where M_1_ reflected all GLP‐1R–positive staining and the neuronal fraction, whilst M_2_ reflected all neuronal staining and the fraction that was GLP‐1R–positive. To account for size differences of the ganglia, the co‐localisation values were normalised to the total area of each ganglion. For negative control images, the M1 & M2 coefficients were ‘0’ using both thresholds.

### 2.5 Statistical Analysis

Each anatomical region had five different patients; for each patient, five fields of view were used. For each patient per group, the average of the fields of view was used. Therefore, each group had a total of five data points determined from the average of the five fields of view per patient. Mean number of positive pixels and Mander's overlap between antibody stains were performed by ordinary one‐way analysis of variance (ANOVA) followed by Tukey's post hoc test to compare between groups. All data distributions were assessed for normality using the Shapiro–Wilk test before parametric analyses, and all data were normally distributed (Gaussian), unless stated. Values are expressed as mean ± standard error of the mean (SEM). GraphPad Prism 10 was used for statistical analysis (RRID:SCR_002798). Statistical significance was set at *p* < 0.05. No test for outliers was included; no data points were excluded, and image analysis was not blinded. Sample size calculations were not performed a priori; sample sizes were determined based on prior studies of a similar nature (Bauset et al. 2024; Gill et al. 2008; Takeshita et al. 2006). The data that support the findings of this study are available upon reasonable request to the corresponding author, M.P.

## 3 Results

### 3.1 GLP‐1R Is Expressed on Neuronal Endings Lining the Mucosa

**FIGURE 1:**
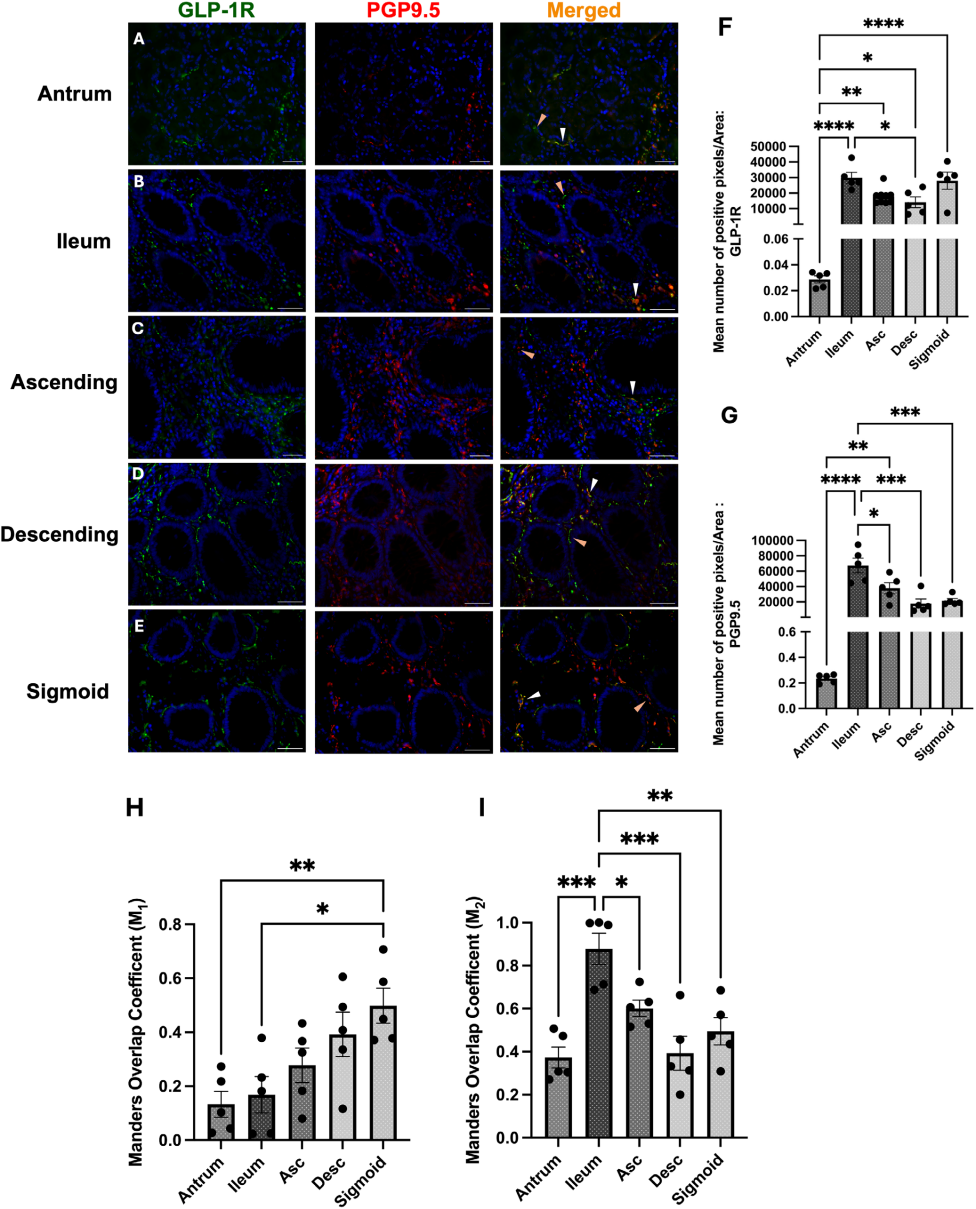
GLP‐1R is more abundant on nerve endings innervating the mucosa of the human lower GI tract. Representative images of GLP‐1R (green) immunoreactivity, co‐localised with neuronal fibres stained with PGP9.5 (red), demonstrating GLP‐1R expression on nerve endings (merged signal in orange). White arrows highlight colocalisation, whilst orange arrows represent a lack of colocalisation. (A) Representative images from the antrum, (B) ileum, (C) ascending colon, (D) descending colon and (E) sigmoid colon. (F, G) One‐way ANOVA quantification of GLP‐1R (*F*(4, 24) = 13.45, *p* < 0.0001) and PGP9.5 (*F*(4, 20) = 17.14, *p* < 0.0001) expression with mean number of positive pixels by area of the mucosa. (H, I) Overlap of co‐localisation coefficients (M_1_ and M_2_) of GLP‐1R on PGP9.5‐positive neuronal fibres innervating the mucosa, across the human GI tract using Mander's coefficient (One‐way ANOVA, M_1_: (*F*(4, 20) = 5.3, *p* = 0.0044), M_2_: (*F*(4, 20) = 10.97, *p* < 0.0001)). Scale bar = 50 μm, objective 40×. For each anatomical region, five patients were analysed, with five images acquired per patient. Each data point represents the average of the mean values for each patient, per region. Further statistical information is detailed in Table S3. *p < 0.05, **p < 0.01, ***p < 0.001, ****p < 0.0001.

As the mucosa of the GIT is densely innervated by both extrinsic and intrinsic neurons, mucosal expression of GLP‐1R was assessed and quantified using PGP9.5 to determine the mean number of positive pixels per area of the mucosa for GLP‐1R, PGP9.5 and the colocalisation values, reflective of GLP‐1R identified on neuronal endings (Furness et al. 2014). Positive mucosal GLP‐1R staining was observed from the antrum to sigmoid colon, localised on nerve endings (Figure 1A–E). In the antrum, GLP‐1R‐ and PGP9.5‐expressing fibres were sparse, but where present, co‐localisation was observed (Figure 1A). From the ileum onwards, the mean number of GLP‐1R positive pixels increased significantly, with greater overlap on PGP9.5‐positive fibres (Figure 1B). In the ileum, GLP‐1R immunoreactivity was detected throughout the crypt‐villus axis (Figure 1B). In the colon, GLP‐1R expression was predominantly observed on neuronal fibres along the base of crypts (Figure 1C–E). Quantitatively, the mean number of positive pixels for GLP‐1R significantly increased from antrum to ileum, peaking in the ascending colon and remaining high throughout distal colonic regions (Figure 1F). Colocalisation of GLP‐1R on PGP9.5 positive cells peaked within the ileum and ascending colon (Figure 1I, M_2_; 0.88 and 0.61, respectively).

### 3.2 GLP‐1R Is Expressed in Human Submucosal Plexus

**FIGURE 2:**
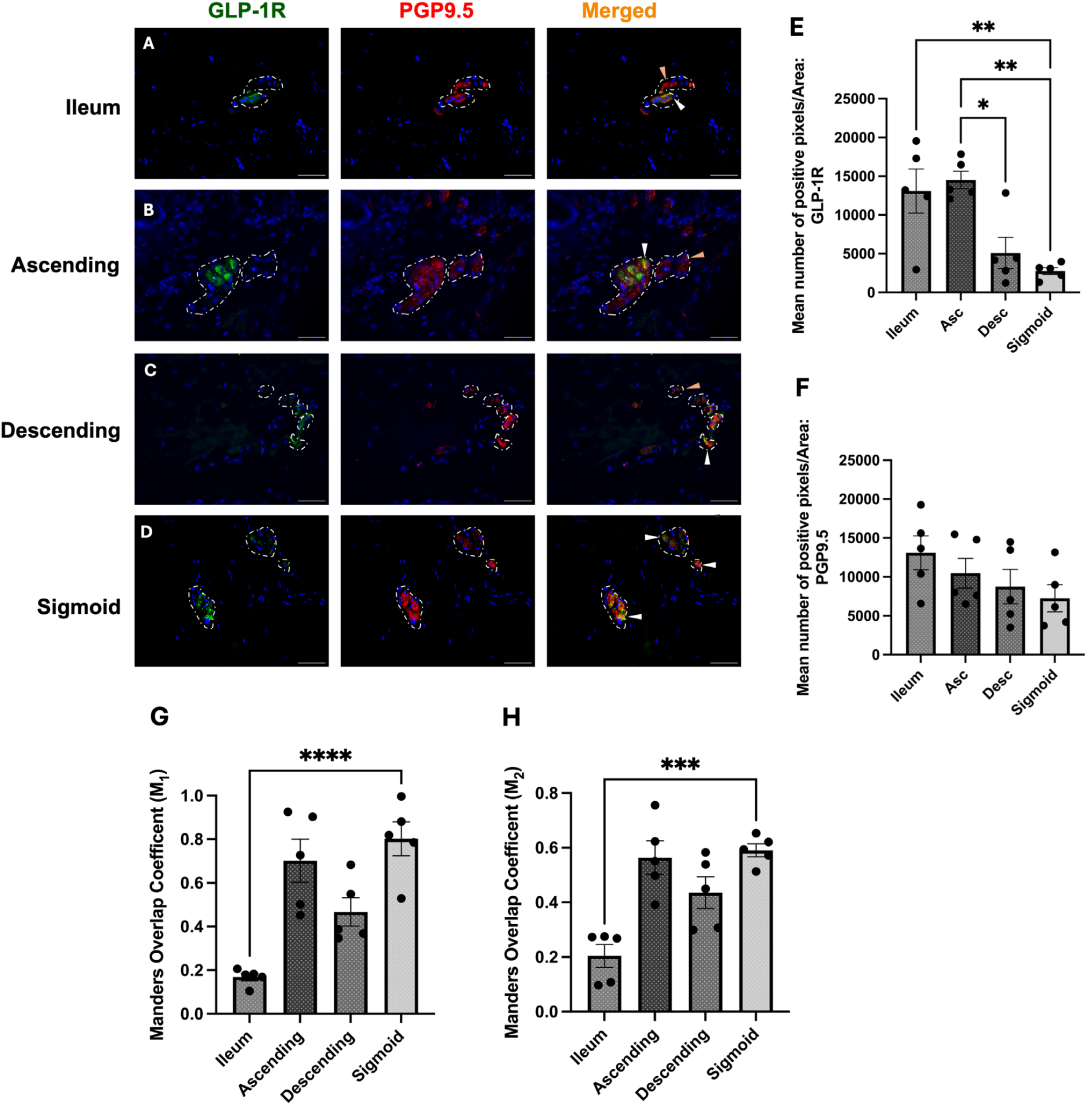
GLP‐1R is expressed in the submucosal plexus throughout the human lower GI tract. Representative images of GLP‐1R (green) immunoreactivity, which co‐localised with PGP9.5 (red), demonstrating GLP‐1R expression on human gut submucosal neurons (merged signal in orange). (A) Representative images from the ileum, (B) ascending colon, (C) descending colon and (D) sigmoid colon. (E, F) Quantification of mean number of positive pixels per area of the submucosal plexus (One way ANOVA, Tukey's Post hoc E; (*F*(3, 16) =9.8, *p* = 0.0006) F; (*F*(3, 16) = 1.54, *p* = 0.2427)). (G, H) Mander's overlap coefficients, M_1_; (*F*(3, 16) = 15.77, *p* < 0.0001) M_2_; (*F*(3, 16) = 13.2, *p* = 0.0001). Scale bar = 50 μm, objective 40×. Dotted lines were used to show the plexus of neurons within the submucosa, determined by the morphology of the tissue, evident with DAPI staining. Images were taken from five different patients per anatomical region. Further statistical information is detailed in Table S3. *p < 0.05, **p < 0.01, ***p < 0.001, ****p < 0.0001.

Expression of GLP‐1R within the submucosal plexus from the ileum to the sigmoid colon was evaluated using immunohistochemistry. The antrum was excluded from analysis because it lacks a submucosal plexus (Furness 2012). Positive GLP‐1R staining was observed in the submucosal plexus of the ileum and all colonic regions. GLP‐1R staining was not present in the surrounding submucosal tissue (Figure 2A–D). Most submucosal neurons showed strong GLP‐1R staining, while some neurons had reduced immunoreactivity (Figure 2B,C). In the submucosal plexuses where co‐localisation of GLP‐1R and PGP9.5 was evident, the staining pattern appeared both cytoplasmic and membrane‐associated (Figure 2B,C). Not all enteric neurons in the submucosal plexus expressed GLP‐1R, as some PGP9.5‐immunoreactive cells were negative for GLP‐1R (orange arrows, Figure 2B,C). Notably, the descending and sigmoid colon exhibited significantly higher GLP‐1R colocalisation compared to the ileum, yet overall reduced GLP‐1R positive pixels per area of the submucosal plexus, at the distal portions of the colon, despite higher colocalisation overlaps in the same regions (Figure 2E–G). Furthermore, as GLP‐1R expression increased from the small to the large intestine, immunoreactivity was observed throughout the entire area of the plexus (Figure 2C,D).

### 3.3 GLP‐1R Is Consistently Expressed in the Muscle Layers of the GI Tract

**FIGURE 3:**
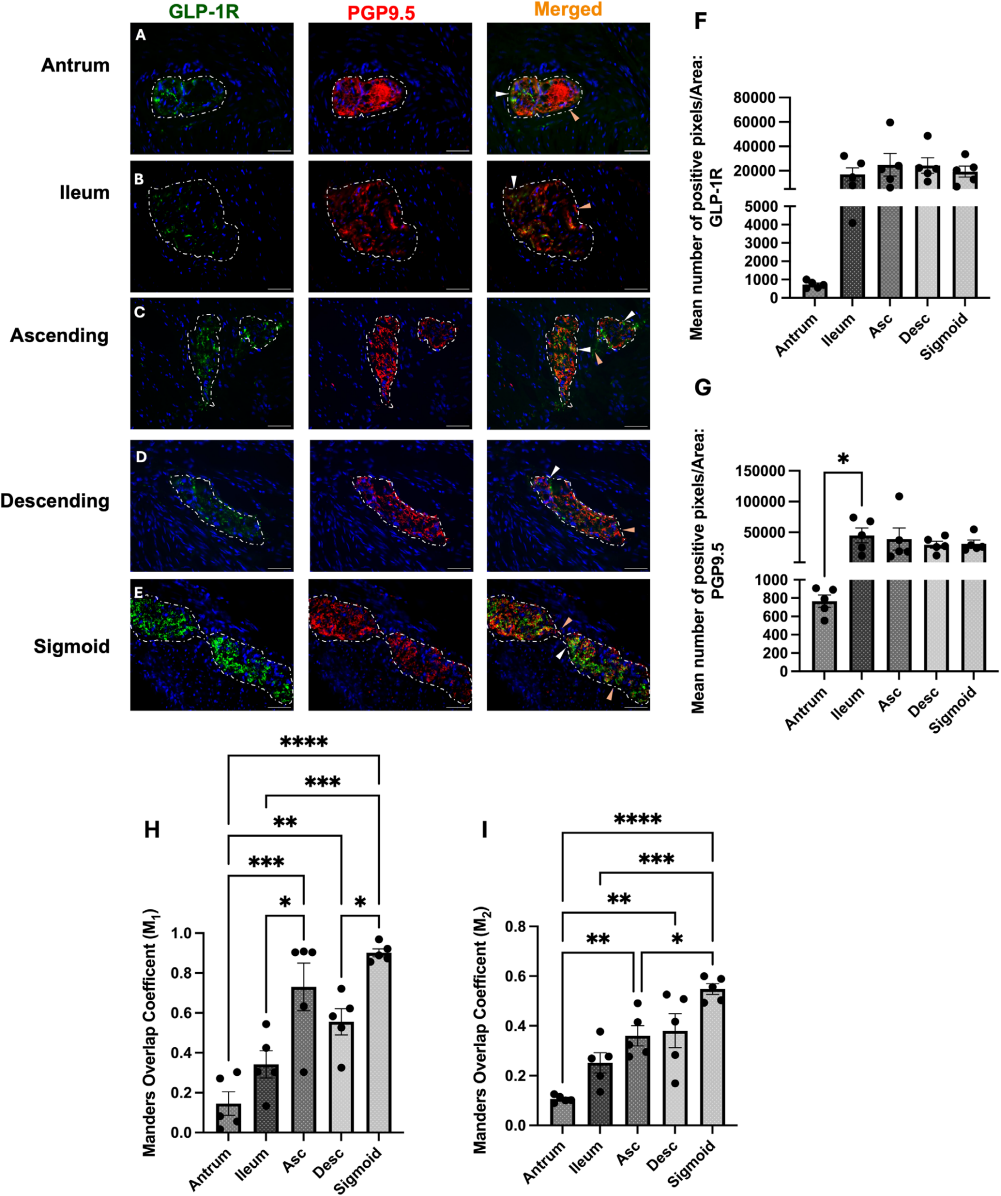
GLP‐1R was expressed throughout the muscle layers of the human GI tract. (A–E) Representative images of GLP‐1R expressed on nerve endings, at the (A) antrum, (B) ileum, (C) ascending, (D) descending and (E) sigmoid colon. Final row from (A–E) represents the co‐localisation of GLP‐1R on PGP9.5‐positive neuronal endings with merged signal (orange). (F, G) Quantification of mean co‐localisation values using Mander's coefficient (F; (*F*(4, 20) = 5.56, *p* = 0.0036), G; (*F*(4, 20) = 1.99, *p* = 0.1347)). (H, I) Mander's overlap coefficients, M_1_; (*F*(4, 20) = 17.66, *p* < 0.0001), M_2_; (*F*(4, 20) = 21.44, *p* < 0.0001). Data are shown as mean ± SEM, representative of five patients per region. Further statistical information is detailed in Table S3. *p < 0.05, **p < 0.01, ***p < 0.001, ****p < 0.0001.

GLP‐1R is known to play a significant role in motility by modulating external musculature. Therefore, GLP‐1 receptor expression was quantified in the muscle layers of the GI tract (Figure 3). Expression was evidently heterogeneous at the level of the colon with clear fibre expression and colocalisation (Figure 3A–E). Colocalisation increased distally throughout the GI tract, with visible colocalisation on nerve endings innervating the muscle (Figure 3E,H,I). Quantitatively, GLP‐1R mean positive pixels increased significantly from the antrum to the ileum, which then reduced at the colon. Interestingly, the colocalisation values increased at the level of the colon (Figure 3G–I).

### 3.4 GLP‐1R Is Expressed in the Human Myenteric Plexus

**FIGURE 4:**
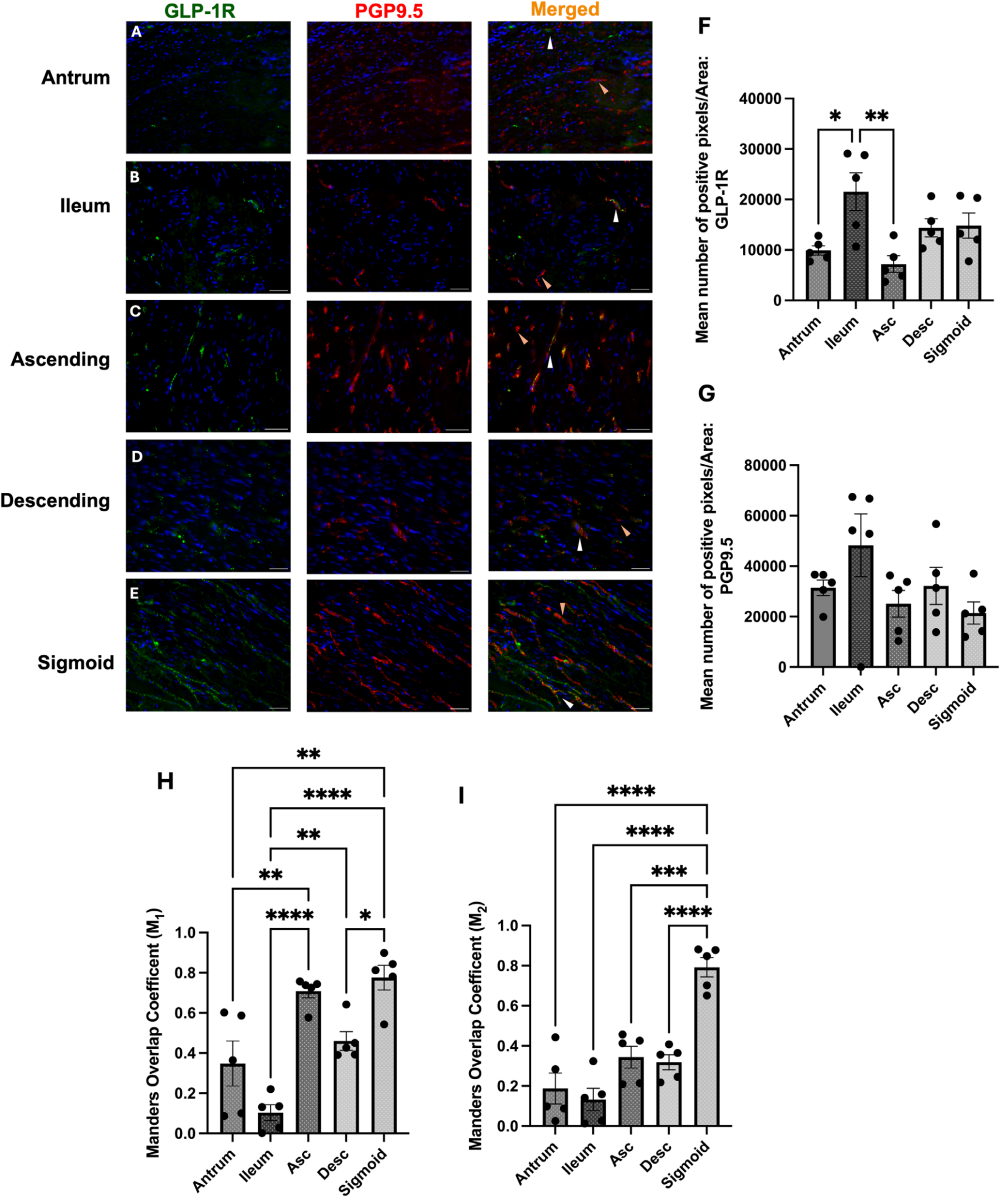
GLP‐1R is expressed within the human myenteric plexus across the upper and lower GI tract. Representative images demonstrating GLP‐1R immuno‐positive cells (green), which co‐localised with PGP9.5 (red) to show myenteric neurons expressing GLP‐1R (orange). Images were taken from the antrum (A), ileum (B), ascending (C), descending (D) and sigmoid colon (E). (F, G) Quantification of GLP‐1R co‐localisation within the myenteric plexus, across GI regions (F; (*F*(4, 20) = 2.74, *p* = 0.0573), G; (*F*(4, 20) = 2.71, *p* = 0.0596)). (H, I) Mander's coefficient was used to quantify GLP‐1 receptor expression on neurons (M_1_; (*F*(4, 20) =16.69, *p* < 0.001), M_2_; (*F*(4, 20) = 15.85, *p* < 0.0001)). Scale bar = 50 μm, objective 40×. Dotted lines were used to show the myenteric plexus within the muscle layers determined by the morphology of the tissue, evident with DAPI staining. Images were taken from five different patients. Further statistical information is detailed in Table S3. * *p* < 0.05, ** *p* < 0.01, *** *p* < 0.001, **** *p* < 0.0001.

GLP‐1R was positively expressed within the myenteric plexuses of all investigated regions of the human gastrointestinal tract. GLP‐1R expression was observed throughout the myenteric plexus (Figure 4A–E). Notably, GLP‐1R expression was restricted to the myenteric neurons, with no detectable staining in the immediate surrounding muscle cells (Figure 4A–E). Punctate GLP‐1R labelling did not colocalise with DAPI‐stained nuclei, suggesting that these immunoreactive structures are terminal varicosities (Figure 4A,C). The degree of immunoreactivity was heterogeneous, where the ileum had a lower number of mean positive GLP‐1R pixels compared to the sigmoid colon (Figure 4B,C,E). Additionally, GLP‐1R immunoreactive staining appeared punctate in the antrum, ascending and sigmoid colon (Figure 4A,C,E). GLP‐1R expression in the ileum and descending colon was more diffuse (Figure 4B,D). Image analysis of GLP‐1R co‐localisation with PGP9.5‐immunoreactive myenteric neurons revealed significantly greater GLP‐1R colocalisation in all lower GIT regions compared to the antrum (Figure 4H,I).

### 3.5 GLP‐1R Is Expressed on Enteric Neuronal Subtypes in Myenteric Plexus of Human Ascending Colon

To identify specific myenteric neuronal subtypes expressing GLP‐1R, ENS excitatory and inhibitory neurons were identified. This was determined via their expression of ChAT and nNOS, respectively. Specifically, nNOS catalyses the production of nitric oxide (NO), the primary inhibitory neurotransmitter in the GI tract, whilst ChAT synthesises acetylcholine (ACh), the primary excitatory neurotransmitter in the GI tract (Unno et al. 2006).

**FIGURE 5:**
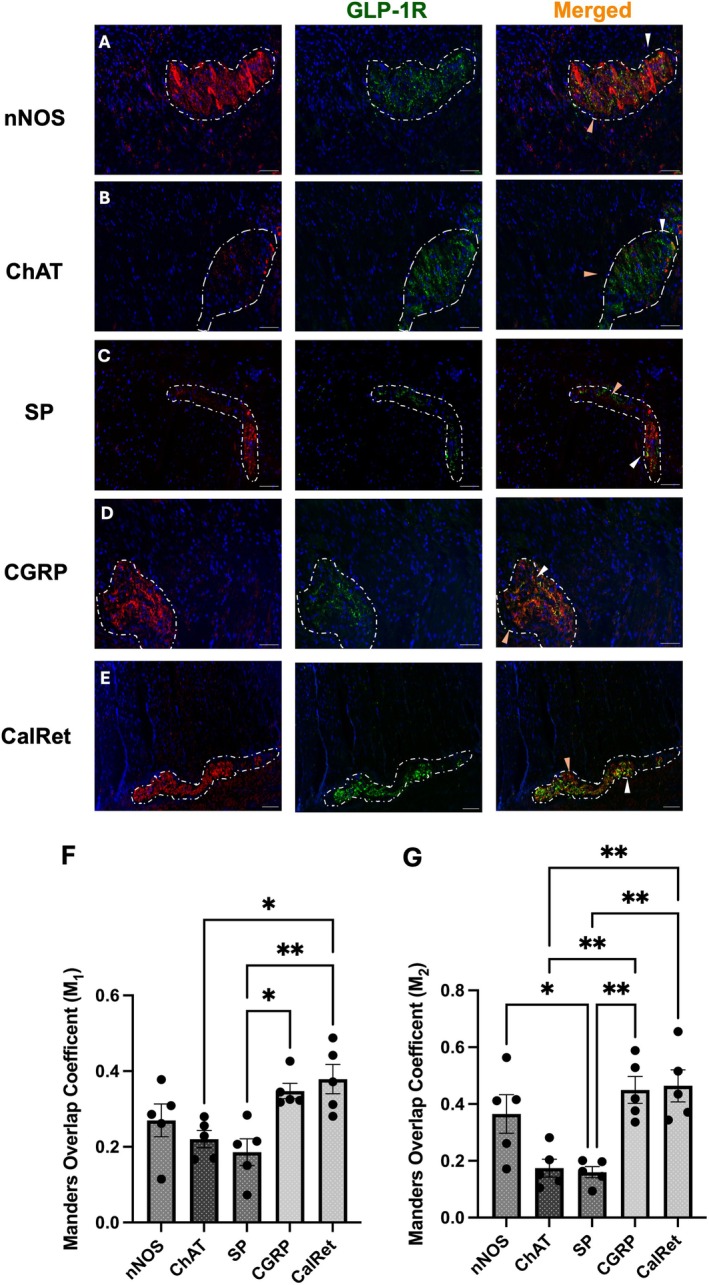
GLP‐1R has a diverse neurochemical co‐localisation pattern found within the myenteric plexus of the human ascending colon. All neuronal markers are shown in red in the figure, where (A) Positive staining with nNOS (nitrergic neurons), (B) ChAT (cholinergic neurons), (C) SP (Substance P neurons), (D) CGRP (Calcitonin gene‐related peptide neurons) and (E) CalRet (Calretinin neurons). The merged signal (orange) represents neurons positive for both GLP‐1R and the respective neuronal marker. nNOS immunoreactivity was determined by a positive signal with a specific antibody recognising neuronal nitric oxide (NO) synthase (nNOS). (F, G) Mander's coefficient was used to analyse and quantify GLP‐1R co‐localisation with neuronal markers (F; (*F*(4, 20) = 6.05, *p* = 0.0023), G; (*F*(4, 20) = 9.48, *p* = 0.0002)). Dotted lines were used to show the myenteric plexus within the muscle layers, evident with DAPI staining. White arrows demonstrate colocalisation, whilst the orange arrows demonstrate no colocalisation. Images were taken from five different patients. Further statistical information is detailed in Table S3. *p 0.05, ** *p* < 0.01, ***p < 0.001, ****p < 0.0001.

GLP‐1R co‐localised with nNOS‐expressing inhibitory neurons and presented a punctate staining pattern (Figure 5A). In contrast, GLP‐1R‐ and ChAT‐expressing neurons appeared punctate rather than cytoplasmic or membranous (Figure 5B). Quantitative analysis using Mander's coefficient revealed significantly greater GLP‐1R co‐localisation in nNOS‐positive neurons compared to ChAT‐positive neurons, with a 2‐fold higher co‐localisation (M_2_: 0.365 vs. 0.174, respectively; Figure 5F).

Substance P (SP)‐expressing neurons, also a neurochemical marker of axonal and terminal processes from multiple enteric neurons, including excitatory motor neurons (Bigalke and Habermann 1980; Li et al. 2014), and SP profiles exhibited sparse GLP‐1R co‐localisation with scattered and punctate staining of variable intensity across the ganglia (Figure 5C). SP‐expressing neurons had the lowest level of GLP‐1R co‐expression among all subtypes analysed, with significantly lower GLP‐1R co‐localisation in SP‐expressing neurons compared to nNOS‐positive neurons (M_1_: mean co‐localisation value was 0.186, M_2_: 0.16) (Figure 5F).

Calcitonin gene‐related peptide (CGRP) is present in multiple enteric neurons, including the varicosities of extrinsic primary afferent neurons (Peiris et al. 2022), and also showed extensive GLP‐1R co‐localisation with dense, punctate staining throughout the plexus (Figure 5D). Among all neuronal subtypes, CGRP‐expressing neurons exhibited the highest GLP‐1R co‐localisation, with a Mander's coefficient (M_1_ and M_2_) of 0.34 and 0.44, respectively, representing a 2.75‐fold greater co‐localisation compared to SP‐positive neurons (Figure 5F).

**FIGURE 6:**
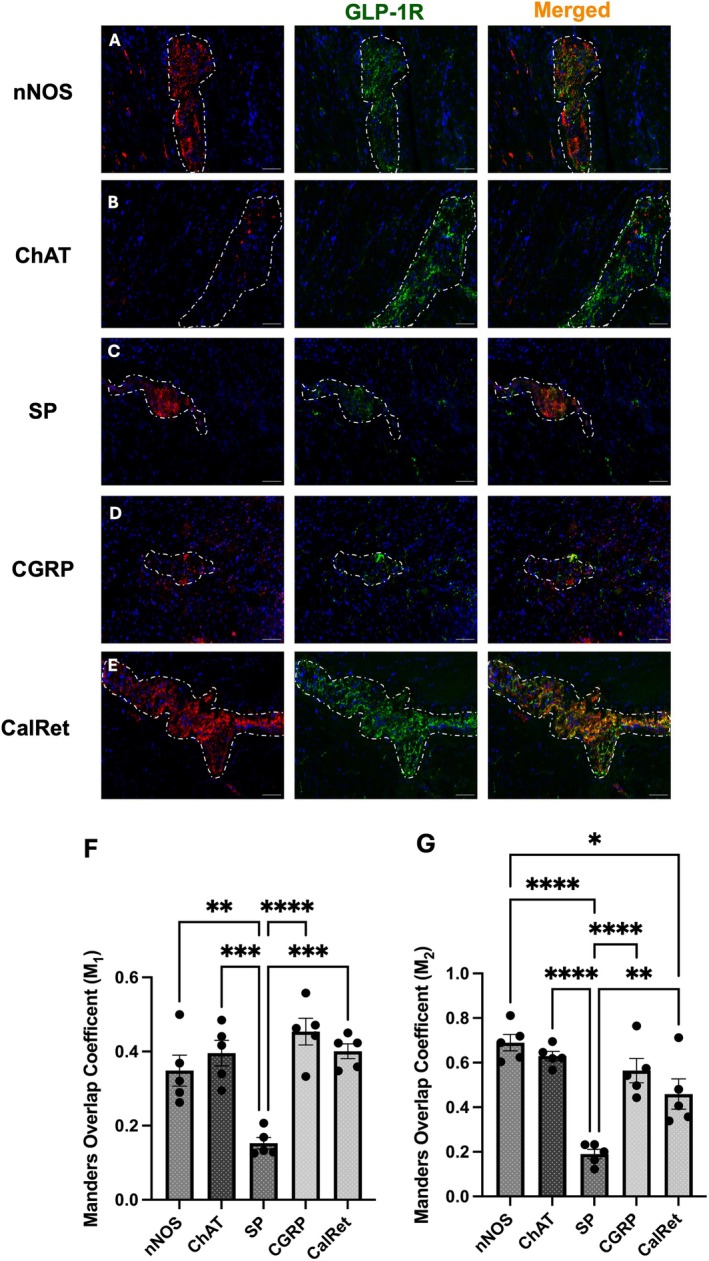
GLP‐1R demonstrated a varied neurochemical coding pattern in the human sigmoid colon with no difference from the ascending colon. (A–E) Representative images of the sigmoid myenteric plexus demonstrating GLP‐1R expressed on various neurons, immunohistochemically identified with positive signals from (A) nNOS, (B) ChAT, (C) SP, (D) CGRP and (E) CalRet. Final row from (A–E) represents the co‐localisation of GLP‐1R on neuronal subpopulations with merged signal (orange). (F, G) Quantification of mean co‐localisation values using Mander's coefficient (F; (*F*(4, 20) = 13.91, *p* < 0.0001), G; (*F*(4, 20) = 19.58, *p* < 0.0001)). Dotted lines were used to show the myenteric plexus within the muscle layers, determined by the morphology of the tissue, evident with DAPI staining. Images were taken from five different patients. Further statistical information is detailed in Table S3. *p < 0.05, **p < 0.01, ***p < 0.001, ****p < 0.0001.

Finally, calretinin showed positive co‐localisation with GLP‐1R and a Mander's coefficient (M_1_ and M_2_) of 0.38 and 0.46, respectively (Figure 5E–G). GLP‐1R‐ and CalRet‐expressing neurons were densely clustered in some regions of the plexus rather than evenly distributed. In some instances where there was no direct co‐localisation, GLP‐1R‐labelled staining was frequently observed adjacent to CalRet‐expressing neurons (Figure 6E).

### 3.6 GLP‐1R Neurochemical Expression Is Consistent Within the Myenteric Plexus of Sigmoid Colon

Sigmoid colon expression of GLP‐1R showed a similar staining and co‐localisation profile to that observed in the ascending colon. GLP‐1R expression was observed in both cholinergic (ChAT) and nitrergic (nNOS) neurons within the myenteric plexus (Figure 6A,B). GLP‐1R was expressed in nNOS‐expressing neurons, displaying a punctate co‐localisation pattern throughout the myenteric plexus (Figure 6A) similar to the co‐expression observed with ChAT‐positive neurons, which was also predominantly punctate (Figure 6B). Consistent with findings in the ascending colon, the proportion of GLP‐1R expressed on nNOS‐labelled neurons was higher than that on ChAT‐expressing neurons but did not reach statistical significance (Figure 6A,F).

Substance P (SP)‐expressing excitatory motor neurons in the sigmoid colon exhibited the lowest levels of GLP‐1R co‐localisation among all subtypes examined. Quantitative analysis revealed a Mander's coefficient (M_2_) of 0.19 for SP and GLP‐1R co‐expressing neurons. Although GLP‐1R expression in SP‐immunoreactive neurons was slightly higher in the sigmoid colon compared to the ascending colon, the difference was not statistically significant (mean co‐localisation = 0.15 (M_1_), 0.19 (M_2_)).

Calretinin‐expressing neurons also exhibited GLP‐1R expression throughout the myenteric plexus (Figure 6E). Similar to the ascending colon, neurons which were immunoreactive for CalRet and GLP‐1R did not have a uniform distribution across the plexus.

Lastly, GLP‐1R staining was prominent in CGRP‐immunoreactive nerve endings located within the peripheral muscle layers surrounding the myenteric plexus—a notable difference from the ascending colon, where CGRP‐positive endings were largely absent from the muscular layers (Figure 5D vs. Figure 6D). Qualitatively, GLP‐1R‐immunoreactive cells appeared more abundant within the longitudinal muscle layer compared to the circular muscle and had greater co‐localisation with CGRP‐expressing nerve fibres of the muscle (Figure 6D). CGRP‐expressing neurons showed the highest degree of GLP‐1R co‐localisation compared to all neuronal markers assessed in the sigmoid colon (M_1_ = 0.45 and M_2_ = 0.56; Figure 6F,G). CGRP‐immunoreactive neurons demonstrated nearly complete GLP‐1R co‐localisation, with staining presenting a punctate, scattered pattern throughout the ganglia (Figure 6D).

### 3.7 GLP‐1R Is Expressed on HuC/D Positive Cells Within the Myenteric Plexus

**FIGURE 7:**
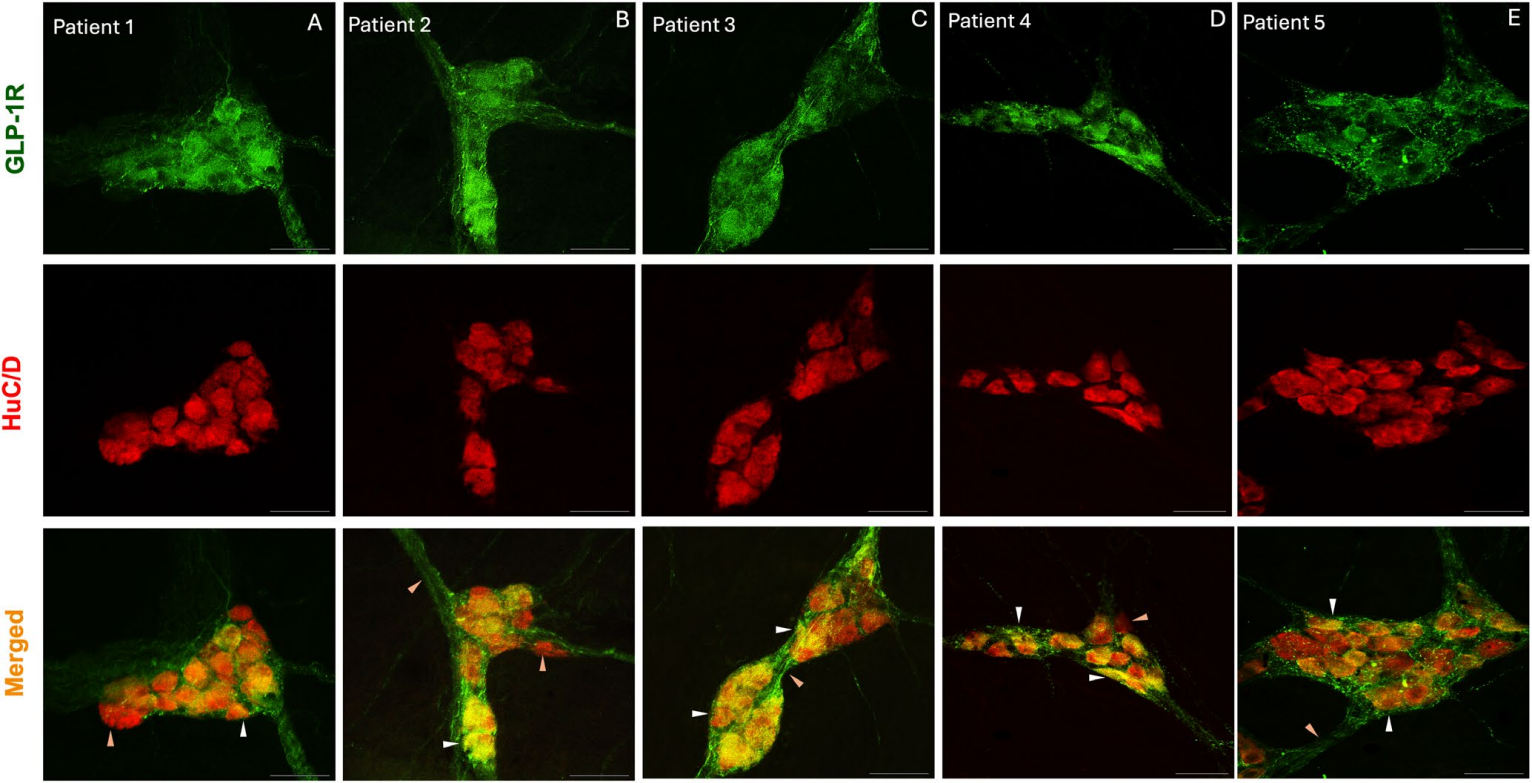
GLP‐1R is expressed on neuronal cell bodies of the myenteric plexus at the sigmoid colon. Representative immunohistochemical images taken from whole mount isolated myenteric plexus in the sigmoid colon, labelled with GLP‐1R (green) and neuronal marker HuC/D (red). Images were taken from five different patients. GLP‐1R colocalised with HuC/D in neurons of the cell body. Representative images are individual optical sections from a Z‐stack image—step = 0.34 μm. Images captured at 40× objective, scale bar (A–F) = 50 μm. Patient information found in Table S2.

Whole‐mount preparations from the sigmoid colon were used to assess the neuronal cell body expression of GLP‐1R. Samples from the sigmoid colon of patients (*N* = 5) were dissected at the taenia and stained for GLP‐1R and HuC/D, a pan‐neuronal marker of cell bodies. GLP‐1R was expressed on a subset of HuC/D‐positive neuronal cell bodies in isolated human colonic myenteric ganglia (Figure 7). Furthermore, GLP‐1R was membrane‐associated, with expression surrounding the myenteric nerve cell bodies (Patients 2, 3 and 5). The GLP‐1R staining pattern was punctate, with no background staining. GLP‐1R presents a clear staining in varicosities outside of the enteric cell body, marked by HuC/D (Patients 2 and 5). This was emphasised in larger ganglia and was less visible in smaller clusters of neurons (Patient 2 compared to Patient 4).

## 4 Discussion

We provide a systematic characterisation of GLP‐1 receptor expression on enteric neuronal structures across the human gastrointestinal tract. Using full‐thickness resected samples from healthy individuals, our data show GLP‐1R expression from the mucosa through to the muscle. Staining was region‐dependent with increased expression from the ileum to sigmoid colon, suggesting potential physiological roles for GLP‐1 in gut function, beyond those previously described (Symonds et al. 2015; Peiris et al. 2022; Cani et al. 2009; Baumard et al. 2021; Greiner et al. 2024). Our characterisation data is timely, as the use case of GLP‐1 agonists increases globally; there is a need to understand the functional actions of GLP‐1 not only in mediating satiety, but also its role in gut physiology (Nauck et al. 2021).

In mucosal fibres, receptor abundance increased proximally from the stomach, in parallel with the distribution of L‐cells, which store and secrete GLP‐1, PYY and OXM, that increase distally (Gribble and Reimann 2016; Peiris et al. 2022; Eissele et al. 1992; Baumard et al. 2021; Bellono et al. 2017; Billing et al. 2018). Our observations are consistent with previous reports of mucosal receptor expression, both in mouse proximal colon and human stomach (Symonds et al. 2015; Peiris et al. 2022). However, we found no evidence of nuclear localisation within glandular epithelial (parietal) cells (Broide et al. 2013), instead, we observed staining along neuronal endings often in proximity to the base of colonocytes. As L‐cells are specialised colonocytes, our data support a model whereby GLP‐1 released from L‐cells is likely to activate GLP‐1R expressed within the human mucosa and submucosal plexus and thus regulate absorption and secretory functions. This is further supported by the presence of L‐cell neuropods, basal extensions containing vesicles that have been shown to juxtapose nerve endings, albeit their prevalence remains debated (Gribble and Reimann 2016; Cao et al. 2024; Gribble and Reimann 2022; Kaelberer et al. 2020; Bohórquez et al. 2011; Liddle 2019). Within the ENS, GLP‐1R staining was detected in both membrane‐associated and cytoplasmic compartments, consistent with a dynamic GPCR undergoing rapid internalisation, as previously shown in HEK293 cells and mouse pancreatic islets (Roed et al. 2014). Moreover, these observations align with Grunddal et al., who observed GLP‐1R localisation predominantly on perisomatic nerve fibres rather than neuronal soma, using a pan‐neuronal marker on murine proximal small intestine and distal large intestine (Holst et al. 2022; Grunddal et al. 2022). The distribution is consistent with a predominantly membrane‐associated GPCR under the conditions examined, while not excluding GLP‐1R internalisation or signalling from intracellular compartments, which were not directly assessed in this study. Importantly, receptor density was greatest in distal regions, suggesting that GLP‐1 may exert greater functional roles in the colon, where L‐cell density increases towards the rectum, than in preceding gut regions (Gunawardene et al. 2011).

In the human distal colon, GLP‐1R localisation showed clear neuronal subtype selectivity within each myenteric plexus. GLP‐1R was not expressed on smooth muscle cells surrounding the plexus, but on nerve fibres innervating the muscle layers. The greatest degree of co‐localisation was observed on CGRP‐positive neurons and nNOS‐positive neurons, with limited co‐localisation on ChAT‐ and substance P‐positive neurons. CGRP‐positive afferents, expressed throughout the myenteric neurons along the GI tract, engage in bidirectional crosstalk with GLP‐1 pathways (Anand et al. 2018; Nakamori et al. 2021). In rodents, GLP‐1 activates CGRP‐containing intrinsic afferents to accelerate colonic peristalsis in the rat colon, whilst long‐acting CGRP analogues increase circulating GLP‐1 by 60% (Anand et al. 2018; Nakamori et al. 2021; Mayer et al. 1990; Sanford et al. 2019). Our findings support similar pathways occurring in the human colon, whereby GLP‐1R expressed on CGRP‐positive neurons could facilitate integration of sensory input with motor and secretory outputs, modulating enteric reflex pathways that govern epithelial secretion and smooth muscle function (May et al. 2019; Bonura et al. 2023).

In addition, preferential localisation to nNOS neurons aligns with prior murine studies demonstrating GLP‐1–mediated inhibition of cholinergic contractions via a nitrergic pathway (Amato et al. 2010, 2014). Clinically, colonic dysmotility is associated with GLP‐1R mimetics such as liraglutide and semaglutide, leading to delayed whole‐gut transit time and constipation (Sharma et al. 2018; Nauck et al. 2021; Kim and Yoo 2025; Cymbal et al. 2025). GLP‐1 influences the inhibitory tone and excitatory drive—pathways that coordinate gastric emptying, smooth muscle relaxation and gut transit times (Tolessa et al. 1998a, 1998b). Furthermore, GLP‐1‐secreting tumours cause severe constipation and delayed colonic transport (Brubaker et al. 2002). The differential neurochemical expression of GLP‐1R presented here begins to delineate the mechanistic underpinning of the collective observations of GLP‐1 activity in the gut and warrants further characterisation via functional experiments.

By contrast, we observed modest co‐localisation of GLP‐1R with Substance‐P expressing neurons in the myenteric plexus. Although Substance P is a conserved excitatory neurotransmitter in intestinal smooth muscle and enteric nerve cells, there is a paucity of quantitative expression data in human lower GI tract (Llewellyn‐Smith et al. 1984). Selective GLP‐1R localisation within inhibitory neurons is consistent with data showing GLP‐1 reduces gut motility, whereas limited expression on SP‐positive neurons suggests minimal direct modulation of excitatory sensory circuits by GLP‐1 (Li et al. 2014; Mayer et al. 1990). This may reflect the limited role of GLP‐1 on excitatory sensory neuronal activity in the myenteric plexus. Extending image analysis to include mucosal and submucosal SP‐expressing neurons will clarify potential roles in secretion and blood flow.

Given the short plasma half‐life of endogenous GLP‐1 (~1–2 min) (Maselli and Camilleri 2021), our findings support the hypothesis that peripherally produced GLP‐1 acts locally within the ENS, exerting more pronounced effects in regions of higher receptor density, particularly the distal colon. Preferential GLP‐1R expression on inhibitory motor neurons and sensory afferents suggests GLP‐1R overstimulation by GLP‐1 mimetics at supraphysiological doses could lead to chronic vagal afferent activation, resulting in nausea, delayed gastric emptying and increased colonic motility, causing bloating and diarrhoea (Nakamori et al. 2021).

This study has several limitations. First, the majority of analyses were performed on fixed frozen tissue sections rather than whole‐mount isolations. Whilst whole‐mount preparations would have allowed more detailed assessment of nuclear morphology and receptor localisation around varicosities, much of this study used banked, fixed tissue, as access to fresh gut tissue is limited. Second, double‐label immunohistochemistry can limit the resolution of GLP‐1R‐positive neurons. Recent work has demonstrated that enteric neurons express several neurochemical combinations (Chen et al. 2023). Identifying the varied network of ENS phenotypes requires multiple protein expression protocols, some of which are not optimal for maintaining morphology of fixed frozen sections. Given the imaging approach used, we cannot precisely delineate nuclear or membrane‐associated receptor localisation. Studies performed in cultured neuronal systems have demonstrated that GPCR distribution can be investigated at higher spatial resolutions and can reveal agonist‐induced trafficking and receptor dynamics (Pelayo et al. 2014; Kirchhofer et al. 2025; Décaillot et al. 2008). Applying similar in vitro approaches in future work would therefore provide important insight into GLP‐1R dynamics. Finally, all surgically resected tissue samples were a minimum of 10 cm away from tumour margins and were macroscopically uninflamed; however, we cannot control the impact on receptor expression resulting from chemotherapy or radiotherapy, nor demographic variables such as BMI, age and sex. Moreover, Type 2 Diabetes Mellitus patients treated with metformin were not excluded from this study as tissue donors. It has been previously shown that metformin treatment increases murine islet expression of GLP‐1R and human umbilical vein endothelial cell (HUVEC) GLP‐1R (Maida et al. 2011; Ke et al. 2017). As such, metformin treatment may influence GLP‐1R expression in the human gut tissue cohorts studied. Nevertheless, the present study adds important characterisation data profiling expression of GLP‐1R across the human GI tract and in all tissue regions, providing a foundation to continue understanding the functional role of GLP‐1 in human gut processes.

Collectively, our findings of mucosal GLP‐1R localisation and distribution within the human GIT provide anatomical data to support GLP‐1 as a key regulator of peripheral pathways modulating gut functions, in addition to its incretin and satiety effects (Furness et al. 2014; Dontamsetti et al. 2024; Peiris et al. 2022; Baumard et al. 2021; Dockray 2003; Raybould 2010). Data presented here show an abundance of GLP‐1R, expressed throughout the human ENS, with increased expression in the colonic region and selective localisation to different neurons. These data extend prior animal studies but importantly provide a mechanistic insight into the observed gastrointestinal side‐effect profile resulting from clinical use of GLP‐1R mimetics. Future studies should address the functional roles of GLP‐1 in regulating gastrointestinal function, particularly secretion and motility in the lower gut, beyond its well‐characterised incretin and appetite‐regulating effects.

## Author Contributions

**Madusha Peiris:** conceptualization, funding acquisition, supervision, resources, methodology, writing – review and editing. **Kiran Devi Dontamsetti:** writing – original draft, data curation, investigation, writing – review and editing, formal analysis, methodology, conceptualization. **Rubina Aktar:** supervision, writing – review and editing.

## Funding

This work was supported by the Medical Research Council [MR/W007045/1].

## Ethics Statement

For human tissue specimens, ethical approval was obtained from the East London and The City HA Local Research Ethics Committee (NREC 09/H0704/2) after written patient consent.

## Conflicts of Interest

The authors declare no conflicts of interest.

## Supporting information


**Figure S1:** AGR‐021 validation tests. (A) Negative control staining for GLP‐1R immunohistochemical imaging. Stained section with the primary antibody omitted in fixed, frozen human GI sections. (B) AGR‐021 blocking peptide was used to stain ascending colon tissue as an additional negative control, where the blocking peptide (in excess, as per the manufacturer's instructions) was combined with the AGR‐021 antibody to the antigen‐binding site. (C) Human pancreatic tissue (tail) was stained with AGR‐021 as a positive control, determined by DAPI morphology, showing a distinct cluster of densely packed nuclei, contrasting with the surrounding pancreatic tissue. Scale bar 50 μm.
**Figure S2:** Representative images taken for the optimisation of antibody dilution. Human GI fixed frozen samples (Ascending colon) were stained with GLP‐1R at dilutions of (A) 1:100, (B) 1:400 or (C) 1:800 to determine the optimal dilution factor for signal‐to‐noise. Scale bar 50 μm.
**Table S1:** Details of 25 patients from whom full‐thickness surgically resected samples were obtained. For all patients listed here, the tissues were used for fixed frozen samples, imaged with epifluorescence microscopy. Asc, ascending colon; Desc, descending colon; Sig, sigmoid colon; F: female; M, male. “‐” refers to no information regarding medications in the records.
**Table S2:** Details of the 5 patients from whom specimens were obtained. These samples were used for whole‐mount dissections to visualise the myenteric plexus with confocal microscopy.
Sig, sigmoid colon; F: female; M: male.
**Table S3:** Summary of Tukey's post hoc comparisons. Individual *p* values following a One‐way ANOVA. Grouped per figure, significance levels correspond to the asterisks denoted in the respective main figures.

## Data Availability

The raw data supporting this work will be available upon reasonable request to the corresponding author.
