# Supplementary material for: Systematic Characterisation of GLP‐1R in Human Enteric Nervous System: Implications for GLP‐1 as a Key Regulator of Colonic Activity
Source: J Neurochem. 2026 May 15;170:e70461. doi: 10.1111/jnc.70461 (PMC13178211; doi:10.1111/jnc.70461)
Supplement: Supplementary file 1 — Figure S1: AGR‐021 validation tests. (A) Negative control staining for GLP‐1R immunohistochemical imaging. Stained section with the primary antibody omitted in fixed, frozen human GI sections. (B) AGR‐021 blocking peptide was used to stain ascending colon tissue as an additional negative control, where the blocking peptide (in excess, as per the manufacturer's instructions) was combined with the AGR‐021 antibody to the antigen‐binding site. (C) Human pancreatic tissue (tail) was stained with AGR‐021 as a positive control, determined by DAPI morphology, showing a distinct cluster of densely packed nuclei, contrasting with the surrounding pancreatic tissue. Scale bar 50 μm. Figure S2: Representative images taken for the optimisation of antibody dilution. Human GI fixed frozen samples (Ascending colon) were stained with GLP‐1R at dilutions of (A) 1:100, (B) 1:400 or (C) 1:800 to determine the optimal dilution factor for signal‐to‐noise. Scale bar 50 μm. Table S1: Details of 25 patients from whom full‐thickness surgically resected samples were obtained. For all patients listed here, the tissues were used for fixed frozen samples, imaged with epifluorescence microscopy. Asc, ascending colon; Desc, descending colon; Sig, sigmoid colon; F: female; M, male. “‐” refers to no information regarding medications in the records. Table S2: Details of the 5 patients from whom specimens were obtained. These samples were used for whole‐mount dissections to visualise the myenteric plexus with confocal microscopy. Sig, sigmoid colon; F: female; M: male. Table S3: Summary of Tukey's post hoc comparisons. Individual p values following a One‐way ANOVA. Grouped per figure, significance levels correspond to the asterisks denoted in the respective main figures. [file JNC-170-e70461-s001.docx]

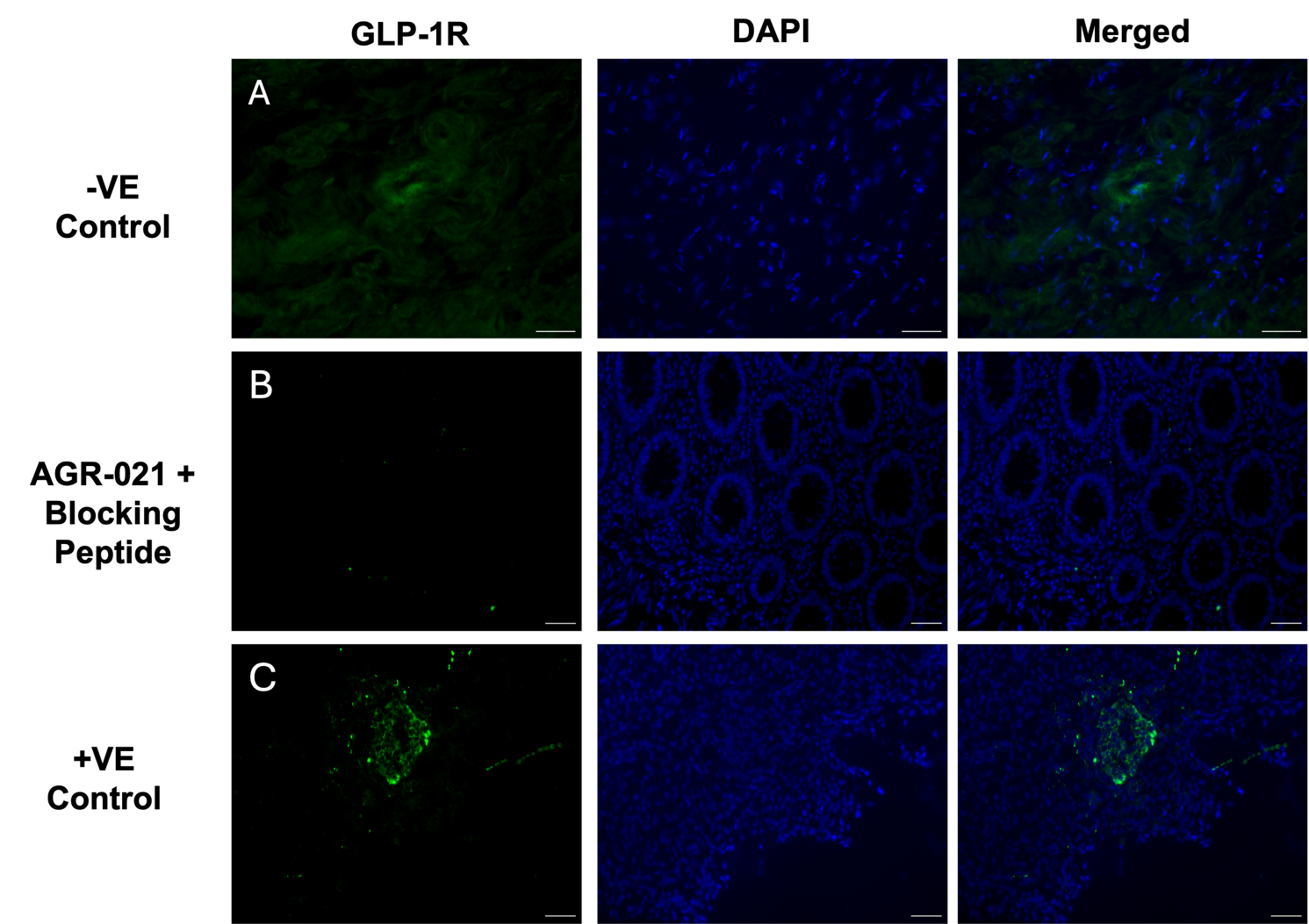


**Fig S1|** AGR-021 validation tests. **A)** Negative control staining for GLP-1R immunohistochemical imaging. Stained section with the primary antibody omitted in fixed, frozen human GI sections. **B)** AGR-021 blocking peptide was used to stain ascending colon tissue as an additional negative control, where the blocking peptide (in excess, as per the manufacturer’s instructions) was combined with the AGR-021 antibody to the antigen-binding site. **C)** Human pancreatic tissue (tail) was stained with AGR-021 as a positive control, determined by DAPI morphology, showing a distinct cluster of densely packed nuclei, contrasting with the surrounding pancreatic tissue. Scale bar 50μm.


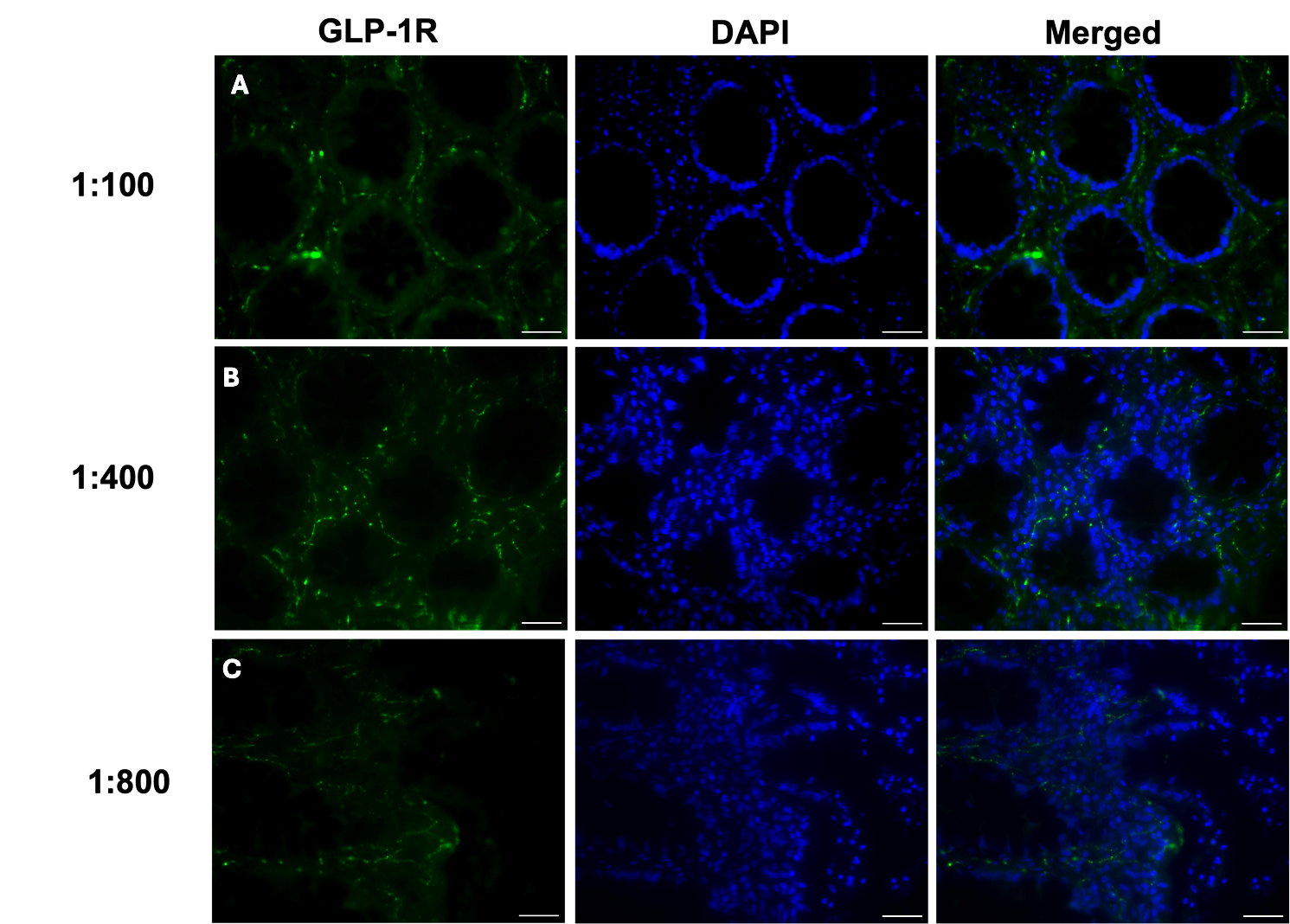


**Fig S2|** Representative images taken for the optimisation of antibody dilution. Human GI fixed frozen samples (Ascending colon) were stained with GLP-1R at dilutions of **(A)** 1:100, **(B)** 1:400, or **(C)** 1:800 to determine the optimal dilution factor for signal-to-noise. Scale bar 50μm.

| **Code** | **Region** | **Diagnosis** | **Surgery** | **Medication** | **Age** | **Sex** |
| --- | --- | --- | --- | --- | --- | --- |
| 16413 | Antrum | Polypoidal mass | Subtotal gastrectomy | - | 67 | M |
| 4913 | Antrum | Gastric carcinoid tumour | Distal subtotal gastrectomy | Metformin | 69 | M |
| 24913 | Antrum | Malignant neoplasm: Pyloric Antrum | Partial gastrectomy and anastomosis of stomach to jejunum NEC | Levomepromazine, co-amoxiclav | 70 | M |
| 191113 | Antrum | Gastric adenocarcinoma | Total gastrectomy | - | 69 | M |
| 281113 | Antrum | Gastric adenocarcinoma | Subtotal gastrectomy | - | 58 | F |
| 2516 | Ileum | Ascending colon cancer | Right hemicolectomy | Amitriptyline, Aspirin, Atorvastatin, Bisoprolol, Empagliflozin, Ferrous Fumarate, Gliclazide, Lansoprazole, Metformin, Ranolazine, | 58 | M |
| 9714 | Ileum | Adenocarcinoma ascending colon | Right hemicolectomy | Amlodipine | 64 | F |
| 22RLH014 | Ileum | Colorectal cancer | Right hemicolectomy | - | 62 | M |
| 22RLH015 | Ileum | Adenocarcinoma of transverse colon | Pan-proctocolectomy | Atorvastatin, Calcichew, Ferrous Fumarate, Gabapentin, Gliclazide, Lansoprazole, Metformin | 64 | F |
| 22RLH029 | Ileum | Caecal mass | Right hemicolectomy | Infliximab, Ustekinumab, | 54 | F |
| 22RLH004 | Asc | Colorectal cancer | Right hemicolectomy | - | 65 | F |
| 22RLH007 | Asc | Colorectal cancer | Right hemicolectomy | - | 58 | M |
| 22RLH028 | Asc | Colorectal cancer | Right hemicolectomy | - | 45 | F |
| 22RLH028 | Asc | Colorectal cancer | Right hemicolectomy | - | 68 | F |
| 22RLH029 | Asc | Colorectal cancer | Right hemicolectomy | - | 70 | F |
| 20415 | Desc | Slow transit constipation | Pan-proctocolectomy | - | 54 | F |
| 23RLH021 | Desc | Colorectal cancer | Anterior resection of rectum | - | 73 | M |
| 41123 | Desc | Colorectal cancer | Anterior resection of rectum | - | 55 | F |
| 230517 | Desc | Rectal polyp | Anterior resection of rectum | Amlodipine, Atenolol, Atorvastatin, Fresubin, Metformin | 59 | F |
| 22RLH015 | Desc | Adenocarcinoma of splenic flexure | Left hemicolectomy | Ramipril, Atorvastatin, Ferrous Fumarate | 55 | F |
| 111214 | Sig | Colorectal cancer | Anterior resection | - | 67 | M |
| 22RLH020 | Sig | Colorectal cancer | Left hemicolectomy | - | 62 | F |
| 22RLH023 | Sig | Colorectal cancer | Anterior resection | - | 57 | M |
| 22RLH024 | Sig | Colorectal cancer | Anterior resection | - | 42 | F |
| 22RLH028 | Sig | Colorectal cancer | Anterior resection | - | 49 | F |

**Table S1|** Details of 25 patients from whom full-thickness surgically resected samples were obtained. For all patients listed here, the tissues were used for fixed frozen samples, imaged with epifluorescence microscopy. Asc, ascending colon; Desc, descending colon; Sig, sigmoid colon; F: female; M, male. “-” refers to no information regarding medications in the records.

| **Code** | **Region** | **Diagnosis** | **Surgery** | **Medication** | **Age** | **Sex** |
| --- | --- | --- | --- | --- | --- | --- |
| 25RLH082 | Sig | T3N, Sigmoid Tumour | Anterior Resection | Piperacillin-tazobactam, fluconazole | 48 | F |
| 25RLH083 | Sig | Rectal Cancer | Anterior Resection | Moviprep, Fresubin, Neomycin, Metronidazole | 51 | M |
| 24RLH003 | Sig | pT1 adenocarcinoma sigmoid polyp | Anterior Resection | Folfirinox | 62 | F |
| 26RLH004 | Sig | Adenocarcinoma 5cm from AV | Anterior Resection | Lansoprazole, Paracetamol, codeine | 57 | M |
| 26RLH0010 | Sig | Adenocarcinoma | Low Anterior Resection | Salbutamol | 69 | M |

**Table S2|** Details of the 5 patients from whom specimens were obtained. These samples were used for whole-mount dissections to visualise the myenteric plexus with confocal microscopy.

Sig, sigmoid colon; F: female; M: male.

|  |  |  |
| --- | --- | --- |
| **Fig 1F** | | |
| **Tukey's multiple comparisons test** | **Summary** | **Adjusted P Value** |
| Antrum vs. Ileum | **** | 0.000011 |
| Antrum vs. Ascending | ** | 0.001839 |
| Antrum vs. Descending | * | 0.041624 |
| Antrum vs. Sigmoid | **** | 0.00003 |
| Ileum vs. Ascending | ns | 0.050134 |
| Ileum vs. Descending | * | 0.017969 |
| Ileum vs. Sigmoid | ns | 0.993451 |
| Ascending vs. Descending | ns | 0.888192 |
| Ascending vs. Sigmoid | ns | 0.129845 |
| Descending vs. Sigmoid | * | 0.045407 |
| **Fig 1G** | | |
| Antrum vs. Ileum | **** | 0.000002 |
| Antrum vs. Ascending | ** | 0.002474 |
| Antrum vs. Descending | ns | 0.276407 |
| Antrum vs. Sigmoid | ns | 0.134666 |
| Ileum vs. Ascending | * | 0.02059 |
| Ileum vs. Descending | *** | 0.000118 |
| Ileum vs. Sigmoid | *** | 0.000304 |
| Ascending vs. Descending | ns | 0.183444 |
| Ascending vs. Sigmoid | ns | 0.357764 |
| Descending vs. Sigmoid | ns | 0.992636 |
| **Fig 1H** | | |
| Antrum vs. Ileum | ns | 0.9952 |
| Antrum vs. Ascending | ns | 0.5476 |
| Antrum vs. Descending | ns | 0.079 |
| Antrum vs. Sigmoid | ** | 0.0071 |
| Ileum vs. Ascending | ns | 0.7708 |
| Ileum vs. Descending | ns | 0.1606 |
| Ileum vs. Sigmoid | * | 0.0164 |
| Ascending vs. Descending | ns | 0.7403 |
| Ascending vs. Sigmoid | ns | 0.1692 |
| Descending vs. Sigmoid | ns | 0.7867 |
| **Fig 1I** | | |
| Antrum vs. Ileum | *** | 0.000111 |
| Antrum vs. Ascending | ns | 0.107155 |
| Antrum vs. Descending | ns | 0.999349 |
| Antrum vs. Sigmoid | ns | 0.642451 |
| Ileum vs. Ascending | * | 0.0366 |
| Ileum vs.  Descending | *** | 0.000184 |
| Ileum vs. Sigmoid | ** | 0.002494 |
| Ascending vs.  Descending | ns | 0.162411 |
| Ascending vs. Sigmoid | ns | 0.741034 |
| Descending vs. Sigmoid | ns | 0.77355 |
| **Fig 2E** | | |
| Ileum vs.  Ascending | ns | 0.9455 |
| Ileum vs.  Descending | * | 0.0344 |
| Ileum vs. Sigmoid | ** | 0.0057 |
| Ascending vs.  Descending | * | 0.0115 |
| Ascending vs. Sigmoid | ** | 0.0019 |
| Descending vs. Sigmoid | ns | 0.8075 |
| **Fig 2F** | | |
| Ileum vs. Ascending | ns | 0.797 |
| Ileum vs Descending | ns | 0.4503 |
| Ileum vs. Sigmoid | ns | 0.2132 |
| Ascending vs. Descending | ns | 0.9298 |
| Ascending vs. Sigmoid | ns | 0.6774 |
| Descending vs. Sigmoid | ns | 0.9513 |
| **Fig 2G** | | |
| Ileum vs. Ascending | *** | 0.000368 |
| Ileum vs. Descending | * | 0.040128 |
| Ileum vs. Sigmoid | **** | 0.000055 |
| Ascending vs. Descending | ns | 0.130488 |
| Ascending vs. Sigmoid | ns | 0.750484 |
| Descending vs. Sigmoid | * | 0.019476 |
| **Fig 2H** | | |
| Ileum vs. Ascending | *** | 0.0004 |
| Ileum vs. Descending | * | 0.0183 |
| Ileum vs. Sigmoid | *** | 0.0002 |
| Ascending vs. Descending | ns | 0.2811 |
| Ascending vs. Sigmoid | ns | 0.9786 |
| Descending vs. Sigmoid | ns | 0.1499 |
| **Fig 3F** | | |
| Antrum vs. Ileum | * | 0.0155 |
| Antrum vs. Ascending | ns | 0.9203 |
| Antrum vs. Descending | ns | 0.6545 |
| Antrum vs. Sigmoid | ns | 0.5766 |
| Ileum vs. Ascending | ** | 0.0025 |
| Ileum vs. Descending | ns | 0.228 |
| Ileum vs. Sigmoid | ns | 0.2798 |
| Ascending vs. Descending | ns | 0.2235 |
| Ascending vs. Sigmoid | ns | 0.1799 |
| Descending vs. Sigmoid | ns | >0.9999 |
| **Fig 3G** | | |
| Antrum vs. Ileum | ns | 0.4951 |
| Antrum vs. Ascending | ns | 0.9707 |
| Antrum vs. Descending | ns | >0.9999 |
| Antrum vs. Sigmoid | ns | 0.8667 |
| Ileum vs. Ascending | ns | 0.2027 |
| Ileum vs. Descending | ns | 0.5369 |
| Ileum vs. Sigmoid | ns | 0.1084 |
| Ascending vs. Descending | ns | 0.957 |
| Ascending vs. Sigmoid | ns | 0.9965 |
| Descending vs. Sigmoid | ns | 0.8351 |
| **Fig 3H** | | |
| Antrum vs. Ileum | ns | 0.096855 |
| Antrum vs. Ascending | ** | 0.006827 |
| Antrum vs. Descending | ns | 0.739757 |
| Antrum vs. Sigmoid | ** | 0.00131 |
| Ileum vs. Ascending | **** | 0.000019 |
| Ileum vs. Descending | ** | 0.007378 |
| Ileum vs. Sigmoid | **** | 0.000004 |
| Ascending vs. Descending | ns | 0.090605 |
| Ascending vs. Sigmoid | ns | 0.946401 |
| Descending vs. Sigmoid | * | 0.020049 |
| **Fig 3I** | | |
| Antrum vs. Ileum | ns | 0.956177 |
| Antrum vs. Ascending | ns | 0.315886 |
| Antrum vs. Descending | ns | 0.48557 |
| Antrum vs. Sigmoid | **** | 0.000002 |
| Ileum vs. Ascending | ns | 0.096671 |
| Ileum vs. Descending | ns | 0.173643 |
| Ileum vs. Sigmoid | **** | <0.000001 |
| Ascending vs. Descending | ns | 0.997526 |
| Ascending vs. Sigmoid | *** | 0.000139 |
| Descending vs. Sigmoid | **** | 0.000069 |
| **Fig 4F** | | |
| Antrum vs. Ileum | ns | 0.3297 |
| Antrum vs. Ascending | ns | 0.0627 |
| Antrum vs. Descending | ns | 0.0716 |
| Antrum vs. Sigmoid | ns | 0.214 |
| Ileum vs. Ascending | ns | 0.8775 |
| Ileum vs. Descending | ns | 0.903 |
| Ileum vs. Sigmoid | ns | 0.9986 |
| Ascending vs. Descending | ns | >0.9999 |
| Ascending vs. Sigmoid | ns | 0.9619 |
| Descending vs. Sigmoid | ns | 0.9739 |
| **Fig 4G** | | |
| Antrum vs. Ileum | * | 0.0468 |
| Antrum vs. Ascending | ns | 0.1059 |
| Antrum vs. Descending | ns | 0.3082 |
| Antrum vs. Sigmoid | ns | 0.2639 |
| Ileum vs. Ascending | ns | 0.9935 |
| Ileum vs. Descending | ns | 0.8346 |
| Ileum vs. Sigmoid | ns | 0.879 |
| Ascending vs. Descending | ns | 0.969 |
| Ascending vs. Sigmoid | ns | 0.9837 |
| Descending vs. Sigmoid | ns | >0.9999 |
| **Fig 4H** | | |
| Antrum vs. Ileum | ns | 0.355278 |
| Antrum vs. Ascending | *** | 0.000149 |
| Antrum vs. Descending | ** | 0.006493 |
| Antrum vs. Sigmoid | **** | 0.000005 |
| Ileum vs. Ascending | * | 0.010305 |
| Ileum vs. Descending | ns | 0.279931 |
| Ileum vs. Sigmoid | *** | 0.000256 |
| Ascending vs. Descending | ns | 0.467359 |
| Ascending vs. Sigmoid | ns | 0.488475 |
| Descending vs. Sigmoid | * | 0.024941 |
| **Fig 4I** | | |
| Antrum vs. Ileum | ns | 0.132783 |
| Antrum vs. Ascending | ** | 0.002497 |
| Antrum vs. Descending | ** | 0.001129 |
| Antrum vs. Sigmoid | **** | 0.000002 |
| Ileum vs. Ascending | ns | 0.363738 |
| Ileum vs. Descending | ns | 0.212204 |
| Ileum vs. Sigmoid | *** | 0.000467 |
| Ascending vs. Descending | ns | 0.996475 |
| Ascending vs. Sigmoid | * | 0.03029 |
| Descending vs. Sigmoid | ns | 0.062517 |
| **Fig 5F** | | |
| nNOS vs. ChAT | ns | 0.8275 |
| nNOS vs. SP | ns | 0.4094 |
| nNOS vs. CGRP | ns | 0.4878 |
| nNOS vs. CalRet | ns | 0.1816 |
| ChAT vs. SP | ns | 0.9468 |
| ChAT vs. CGRP | ns | 0.0898 |
| ChAT vs. CalRet | * | 0.0228 |
| SP vs. CGRP | * | 0.0199 |
| SP vs. CalRet | ** | 0.0045 |
| CGRP vs. CalRet | ns | 0.9609 |
| **Fig 5G** | | |
| nNOS vs. ChAT | ns | 0.0698 |
| nNOS vs. SP | * | 0.0456 |
| nNOS vs. CGRP | ns | 0.7203 |
| nNOS vs. CalRet | ns | 0.5943 |
| ChAT vs. SP | ns | 0.9995 |
| ChAT vs. CGRP | ** | 0.0047 |
| ChAT vs. CalRet | ** | 0.0029 |
| SP vs. CGRP | ** | 0.0029 |
| SP vs. CalRet | ** | 0.0018 |
| CGRP vs. CalRet | ns | 0.9995 |
| **Fig 6F** | | |
| nNOS vs. ChAT | ns | 0.822278 |
| nNOS vs. SP | ** | 0.00212 |
| nNOS vs. CGRP | ns | 0.162504 |
| nNOS vs. CalRet | ns | 0.762933 |
| ChAT vs. SP | *** | 0.000194 |
| ChAT vs. CGRP | ns | 0.686426 |
| ChAT vs. CalRet | ns | 0.999957 |
| SP vs. CGRP | **** | 0.000012 |
| SP vs. CalRet | *** | 0.00015 |
| CGRP vs. CalRet | ns | 0.752155 |
| **Fig 6G** | | |
| nNOS vs. ChAT | ns | 0.870113 |
| nNOS vs. SP | **** | 0.000001 |
| nNOS vs. CGRP | ns | 0.303869 |
| nNOS vs. CalRet | * | 0.011627 |
| ChAT vs. SP | **** | 0.000008 |
| ChAT vs. CGRP | ns | 0.835634 |
| ChAT vs. CalRet | ns | 0.086761 |
| SP vs. CGRP | **** | 0.00007 |
| SP vs. CalRet | ** | 0.002985 |
| CGRP vs. CalRet | ns | 0.467711 |

**Table S3|** **Summary of Tukey’s Post-hoc comparisons.** Individual P-values following a One-way ANOVA. Grouped per figure, significance levels correspond to the asterisks denoted in the respective main figures.
